# Satisfaction with obstetric care in a population of low-educated native Dutch and non-western minority women. Focus group research

**DOI:** 10.1371/journal.pone.0210506

**Published:** 2019-01-31

**Authors:** Ingrid A. Peters, Anke G. Posthumus, Eric A. P. Steegers, Semiha Denktaş

**Affiliations:** 1 Department of Obstetrics and Gynaecology, division of Obstetrics and Prenatal Medicine, Erasmus University Medical Centre, Rotterdam, The Netherlands; 2 Erasmus School of Social and Behavioral Sciences, Clinical Psychology, Erasmus University Rotterdam, The Netherlands; Mayo Clinic Minnesota, UNITED STATES

## Abstract

**Background:**

Low-educated native Dutch and non-western minority women have inadequate access to obstetric care. Moreover, the care they receive lacks responsiveness to their needs and cultural competences. Gaining a deeper understanding of their experiences and satisfaction with antenatal, birthing and maternity care will help to adjust healthcare responsiveness to meet their needs during pregnancy, childbirth and the postpartum period.

**Methods:**

We combined the World Health Organization conceptual framework of healthcare responsiveness with focus group research to measure satisfaction with antenatal, birthing and maternity care of women with a low-educated native Dutch and non-western ethnic background.

**Results:**

From September 2011 until December 2013, 106 women were recruited for 20 focus group sessions. Eighty-five percent of the women had a non-western immigrant background and 89% a low or intermediate educational attainment. The study population was mostly positive about the provided care during the antenatal phas*e*. They were less positive about the other two phases of care. Moreover, the obstetric healthcare systems’ responsiveness in all phases of care (antenatal, birthing and maternity) did not meet these women’s needs. The ‘respect for persons’ domains ‘*autonomy’*, *‘communication’* and ‘*dignity’* and the ‘client orientation’ domain *‘prompt attention’* were judged most negatively.

**Conclusions:**

The study findings give contextual meaning and starting points for improvement of responsiveness in the provision of obstetric care within a multi-ethnic women’s population.

## Introduction

In the Netherlands, care during the antenatal, birthing and postpartum phases is organised as a system of inter-related primary, secondary and tertiary healthcare services. For women without medical risks, care is provided by community midwives (primary healthcare). For women with medical risks, in-hospital care (secondary and tertiary care) is provided by obstetricians. After delivery, most women and their babies receive maternity care at home from a maternity nurse and a community midwife. This system of care is unique in the world. All three phases of care (antenatal, birthing and maternity care) should be equally available for all women, irrespective of their backgrounds. Inadequate access to this care is associated with increased risks of adverse outcomes, including perinatal mortality.[1–8]

Inequalities in these adverse perinatal outcomes are associated with maternal-, child- and healthcare related risk factors.[9] Maternal risk factors can be divided into medical (e.g. obstetric history) and non-medical risk factors (e.g. living in a deprived neighbourhood, low-education, low health literacy, insufficient Native language proficiency and a non-western ethnic minority background).[4,10–12] Also obstetric healthcare organizational factors such as travelling distance to a health care facility and the delivered care itself (for example prenatal screening and instrumental delivery) influence perinatal outcomes.[9, 13]

Previous research from the Netherlands shows that low-educated native Dutch women and non-western ethnic minority women are twice as likely to make inadequate use of obstetric care when compared with their highly educated native Dutch counterparts.[2, 4, 5, 14–17] This may indicate the existence of a mismatch between the needs of these women and the obstetric healthcare system. Previous studies in antenatal and maternity care on patient experience and satisfaction show that women attach importance to the following aspects: a feeling of being cared for (prompt attention), the attitude and sensitivity of healthcare professionals, communication, creativity and attention for culture and ethnicity.[8, 18–20] These aspects were related to the accessibility and timing of obstetric healthcare that influence perinatal outcomes. [9, 13] The above-mentioned aspects are linked to healthcare system responsiveness. Responsiveness refers to how a system facilitates patients to meet their legitimate non-health expectations when they seek healthcare. [21–23] International organizations like the World Health Organization (WHO) and the International Consortium for Health Outcomes Measurements (ICHOM) underline the applicability of responsiveness to measure satisfaction with care. [22, 24, 25] According to the WHO, responsiveness can be divided into eight domains: autonomy, communication, confidentiality, dignity, prompt attention, quality of basic amenities, choice of care provider and access of social support. [22, 23, 25, 26] These are in line with earlier studies in the fields of social science and principles of medical ethics. [25]

A previous quantitative study assessed the responsiveness of obstetric care, and factors influencing this responsiveness during antenatal care and birthing care, in the Netherlands.[27] The researchers found that overall the performance of responsiveness of the obstetric healthcare system was explained more by healthcare and health related issues than by personal characteristics of the patient. The ‘client orientation’ responsiveness category (WHO domains: prompt attention, quality of basic amenities, choice of care provider and access of social support) was judged most negatively.[13] It should be noted, however, that this study was conducted in a native Dutch and medium and highly educated population.

Therefore, in this study we aim to gain a deeper understanding of the responsiveness of the obstetric healthcare system in the Netherlands as experienced by low-educated native Dutch and non-western ethnic minority women. Therefore, we investigated the satisfaction with antenatal, birthing and maternity care by means of the WHO responsiveness concept combined with semi-structured focus group interviews. This qualitative approach makes it possible to elucidate the women’s personal context and to discover patterns in their experiences of the responsiveness performance of healthcare. [28, 29] These insights can then help to improve healthcare responsiveness, which in turn contributes to better pregnancy outcomes.

## Materials and methods

### Respondents

This research focuses on the experiences of women with antenatal, birthing and maternity care living in deprived areas in the city of Rotterdam, the Netherlands. We used purposive sampling to assure that participants in the focus group interviews represented the important target groups of these deprived neighbourhoods (see Table 1 for an overview of their characteristics).

**Table 1:** Respondent characteristics.

Total n = 98 ^1^	N	%
**Age (years)**		
15–19	23	24
20–29	19	19
30–36	22	22
>36	34	35
**Occupation**^2^		
Employed	27	30
Unemployed	42	46
Student	22	24
**Ethnicity**		
Dutch	12	12
Surinamese	14	14
Antillean	14	14
Cape Verdean	19	20
Turkish	11	11
Moroccan	23	24
Other	5	5
**Children**		
nulliparous	34	35
1 to 2	35	36
≥3	29	29
**Educational attainment**		
Low^5^	22	23
Intermediate^6^	62	66
High^7^	10	11
**Years living in the Netherlands** ^3^		
≥ 20 years	30	60
10–20 years	13	26
<10 years	7	14
**Generation**		
Native	12	12
First	51	52
Second	35	26
**Language Proficiency Level** ^4^		
Absent	7	7
Limited	27	29
Good	60	64

1 Exclusion of n = 8 Turkish respondents because of voice recording error.

2Missing n = 7 Occupation

3 Missing n = 1 Years living in the Netherlands

4 Missing n = 4 self-reported Language Proficiency Level

5 Low Educational Attainment: Early childhood education, Primary education, Lower secondary education

6 Intermediate Educational Attainment: Upper secondary education, Post-secondary non-tertiary education.

7 High Educational Attainment: Short-cycle tertiary education, Bachelor’s or equivalent level, Master’s or equivalent level, Doctoral or equivalent level. International Standard Classification of Education (ISCED, 2011)

Rotterdam is the second-largest city of the Netherlands with about 618,500 inhabitants. A former industrial city and still a port city, Rotterdam has a relatively high proportion of low-educated inhabitants, with relatively high levels of unemployment, income segregation and poverty compared to other large Dutch cities. [30, 31] Rotterdam has both attracted and recruited immigrants from all over the world. A high proportion of them are living in the city’s deprived neighbourhoods.

The largest non-western immigrant populations in Rotterdam originate from Turkey (8%), Surinam (8%), Morocco (7%), the Antilles (4%) and Cape Verde (2%).[32, 33] We made sure to include in the study representatives of all these groups. We included women meeting the following criteria: (1) native Dutch or first, second or third generation ethnic minority status other than the aforementioned countries, (b) pregnant or having children, (c) aged between 15 and 65 years and (d) preferably low educated [defined as childhood education, primary education, lower secondary education, upper secondary education, post-secondary non-tertiary education (ISCED, 2011)]. We included adolescents because of a higher prevalence of teenage pregnancies in Rotterdam in comparison with other large cities in the Netherlands, especially among Antillean and Surinamese adolescents.[34] We included the (grand)mothers because of their experiences with Dutch obstetric care in the past. We could thus investigate possible parallels between their experiences and that of the current reproductive population. The inclusion criteria outlined above were decided upon in consultation with a midwifery researcher and community health workers based in deprived areas in Rotterdam. Moreover, these field experts were involved in the recruitment of the women, and they were consulted in the interpretation of the results.

### Recruitment strategy

We employed active recruitment methods, including by ‘verbal advertising’ and through social networks [35–37]. Firstly, the primary researcher visited: (a) peer education meetings organised by the community health workers, (b) primary schools during coffee breaks, (c) secondary schools and a community college during health(care) educational lessons and (d) neighbourhood community centres. Participants recruited through these methods were asked to connect us with other interested women (‘snow ball sampling’ strategy).[38]

### Focus group interviews

We conducted 20 semi-structured focus-group interviews with 18 women with a Turkish background, 19 women with a Moroccan background, 10 women with a Surinamese background, 12 women with an Antillean background, 20 women with a Cape Verdean background, 12 women with a native Dutch background, and 9 adolescent and 6 pregnant women with various ethnic backgrounds. Each focus group consisted of participants with the same ethnic background, including adolescents and pregnant women. This is a strategy that creates group cohesion, which improves the quality of the discussion. [29]

### Focus group methodology

The locations of the focus group sessions were chosen by the participants. Sometimes it was a private home and sometimes a meeting room of a primary school or community centre. At the beginning of each session, we gave the respondents information about the study, the structure of the session and the use of collected data. We explained to the respondents that we were interested in their experiences and satisfaction with antenatal, birthing and maternity care. We also made clear that any identifying information from the focus group sessions would not be transcribed. The sessions lasted on average 80 minutes. With the permission of the respondents, all sessions were tape-recorded. The first author and two public health experts conducted the interviews. All interviews were conducted in Dutch. Assistance was available for respondents with a limited Dutch language proficiency. If needed the aforementioned field experts and one of the moderators could help to interpret (languages: Papiamento, Turkish, Moroccan Berber, Portuguese and Moroccan Arabic) and further explain questions asked by the moderator.

To verify if we had succeeded in our purposive sampling, we asked all respondents to fill in a short questionnaire about their background [age, ethnic minority status– 1^st^, 2^nd^, 3^rd^ generation, marital or relational status, gestational age in weeks (if applicable), number of prior pregnancies (if applicable), number of children (if applicable), educational attainment and self-reported Dutch language proficiency]. The interview protocol [see S1 Table.] focused on care related experiences based on the domains of responsiveness of care as shown in Box 1.[26, 39–41] During the course of a focus group session the chronology of the interview protocol could be slightly adapted based on the respondents’ preferred topics of discussion.

**Box 1:** The eight responsiveness domains, description and subdomains.

***Respect for persons***			
	*Domain*	*Description*	*Subdomains*
	Autonomy	Involvement in decisions	- Informed decision making - Freedom to reject examination or treatments - Asked permission before testing or starting treatment



	Communication	Clarity of communication	- Understandability of verbal information - Understandability of written information - Encouragement to ask questions - Time to ask questions


	Confidentiality	Confidentiality of personal information	- Confidentiality of conducted consultations - Confidential handling of personal information / data - Confidential handling of medical information / data


	Dignity	Respectful treatment and communication	- Respect of privacy during examinations - Privacy in the examination rooms - Respect from the healthcare provider

***Client orientation***			
	*Domain*	*Description*	*Subdomains*
	Prompt attention	Convenient travel and short waiting times	- Prompt attention of healthcare service - Waiting time after asked for help - Accessibility by phone - Travel time to healthcare service

	Quality of basic amenities	Surroundings	- Quality of hygiene of examination rooms and sanitary facilities - Quality of surroundings (i.e. space, seating, fresh air)

	Choice	Choice of health care provider	- Choice of healthcare provider - Choice of healthcare service - Continuity of healthcare provider - Choice of place of delivery

	Access to family and community support	Contact with outside world and maintenance of regular activities	- Facilitating social support - Consideration home situation when planning an appointment

World Health Organization’s responsiveness concept (2003) ^[23]^			

### Informed consent and ethics

The legal use of the anonymized background data and the voice recording of the focus group sessions were based on verbal informed consent of all participants. Also verbal consent was obtained from the parents of the minors included in the study via the community health workers who contacted the minor. In our opinion verbal consent by the participant, given and recorded at the beginning of the interview to be the most appropriate manner within the low literate study population. Furthermore written consent may be seperated from the actual recording, whilst the consent on the recording itself will not. Based on consultation of the Medical Ethical Committee of the Erasmus Medical Centre, the study meets the standards of the Dutch Central Committee on Research Involving Human Subjects (CCMO) for research that is not included in the scope of the Medical Research Involving Human Subjects Act (WMO).[42] For privacy reasons we altered the names of the women in the illustrative quotes provided in the Results section of this article.

### Data analyses

The audiotaped data of the focus groups were transcribed verbatim. We used the observational notes of the sessions about non-verbal communication and general impressions of the respondents.

**Fig 1:**
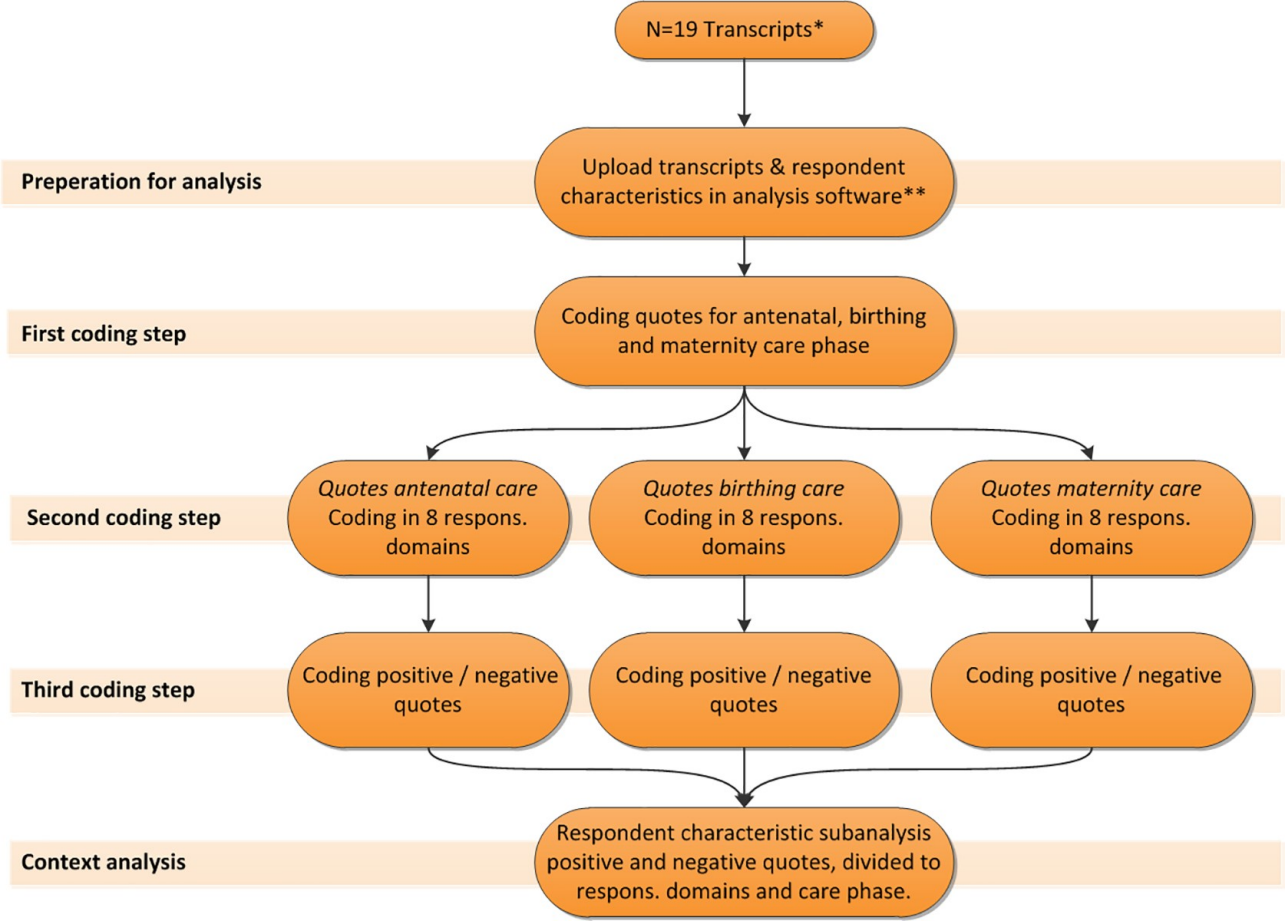
Focus group data analysis. * n = 1 transcript was excluded from the study because of a voice recording error. ** Nvivo 11 Pro Windows. Respons.: responsiveness.

Next, the first author and a group of experts consisting of midwifery researchers, public health experts and field experts, checked all transcripts on interpretation of the audiotaped data. Fig 1 shows the three steps of the coding process. The first step consisted of assigning all quotes to a relevant care phase (antenatal, birthing or postpartum). In the second step, all quotes were coded for the relevant WHO responsiveness domains. For this step we applied the directed content analysis approach in which key concepts from the literature were used to create codes for the analysis.[43] In the third step, all quotes were labelled as either positive or negative. A quote was defined as positive when it contained words such as “good”, “adequate”, “nice” and “sufficient”. A quote was interpreted as negative when it contained words or phrases such as “bad”, “inadequate”, “not sufficient” and “not nice”.

Furthermore, all positively and negatively coded quotes were merged into one dataset, and sub classified according to the phase of care (antenatal care, birthing care and maternity of care), the WHO domains of responsiveness and the background characteristics of the focus group respondents.

Lastly, we conducted a variable-oriented analysis. In this analysis particular variables (such as ethnicity of the respondents) were investigated on their value as a determinant for experienced care during the antenatal, birthing and postpartum phases. [44] Individual level background characteristics were linked to the transcribed quotes. This analysis was performed with the qualitative analysis software Nvivo 11 Pro Windows.

## Results

**Fig 2:**
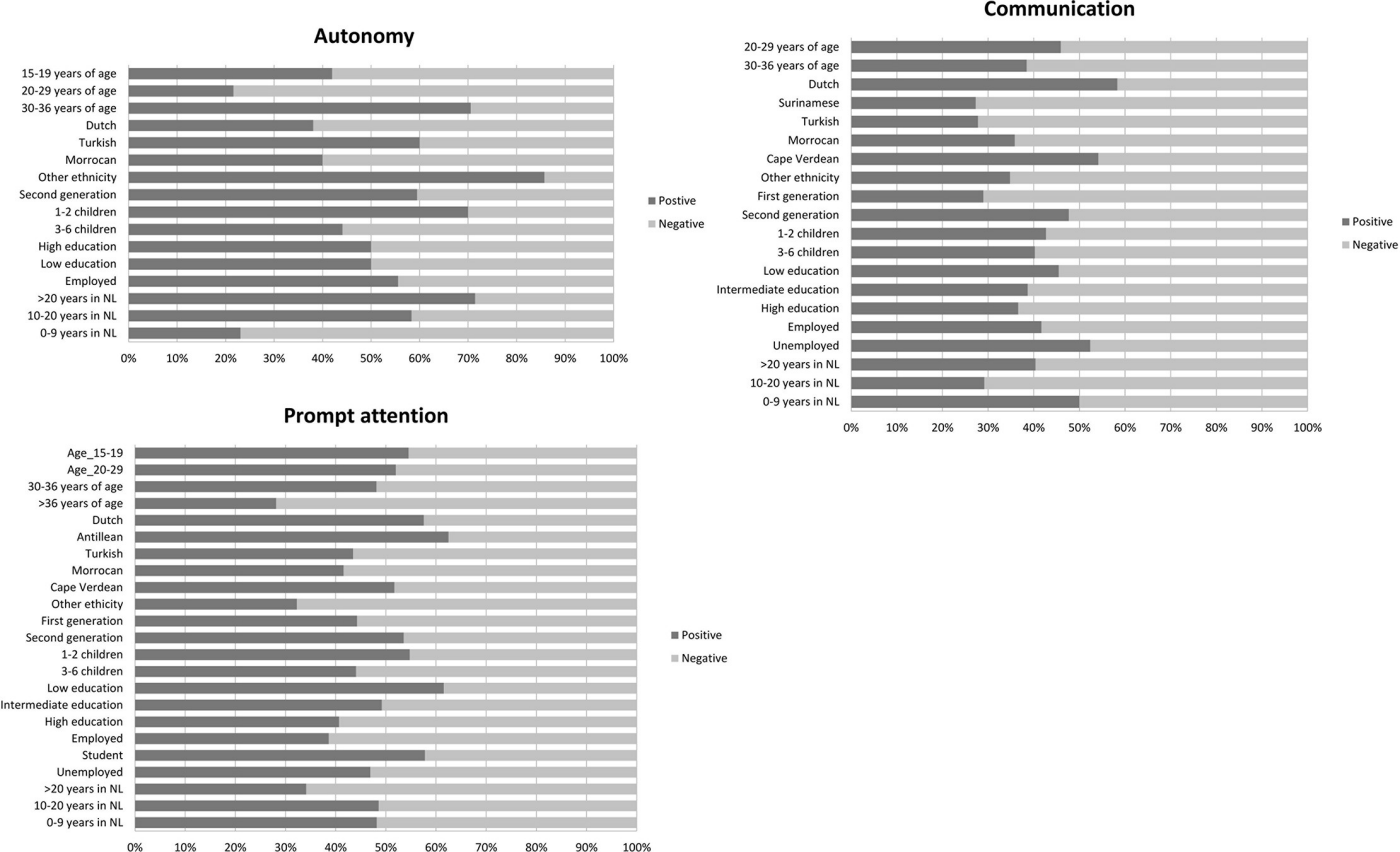
Revelant responsiveness domains in antenatal care. If the majority of the respondents voiced their opinion on a specific domain, the results (either positive or negative) were presented in this figure. Positive: a quote was defined as positive when words such as “good”, “adequate”, “nice” and “sufficient” were used. Negative: a quote was interpreted as negative when words such as “bad”, “inadequate”, “not sufficient” and “not nice” were used.

**Fig 3:**
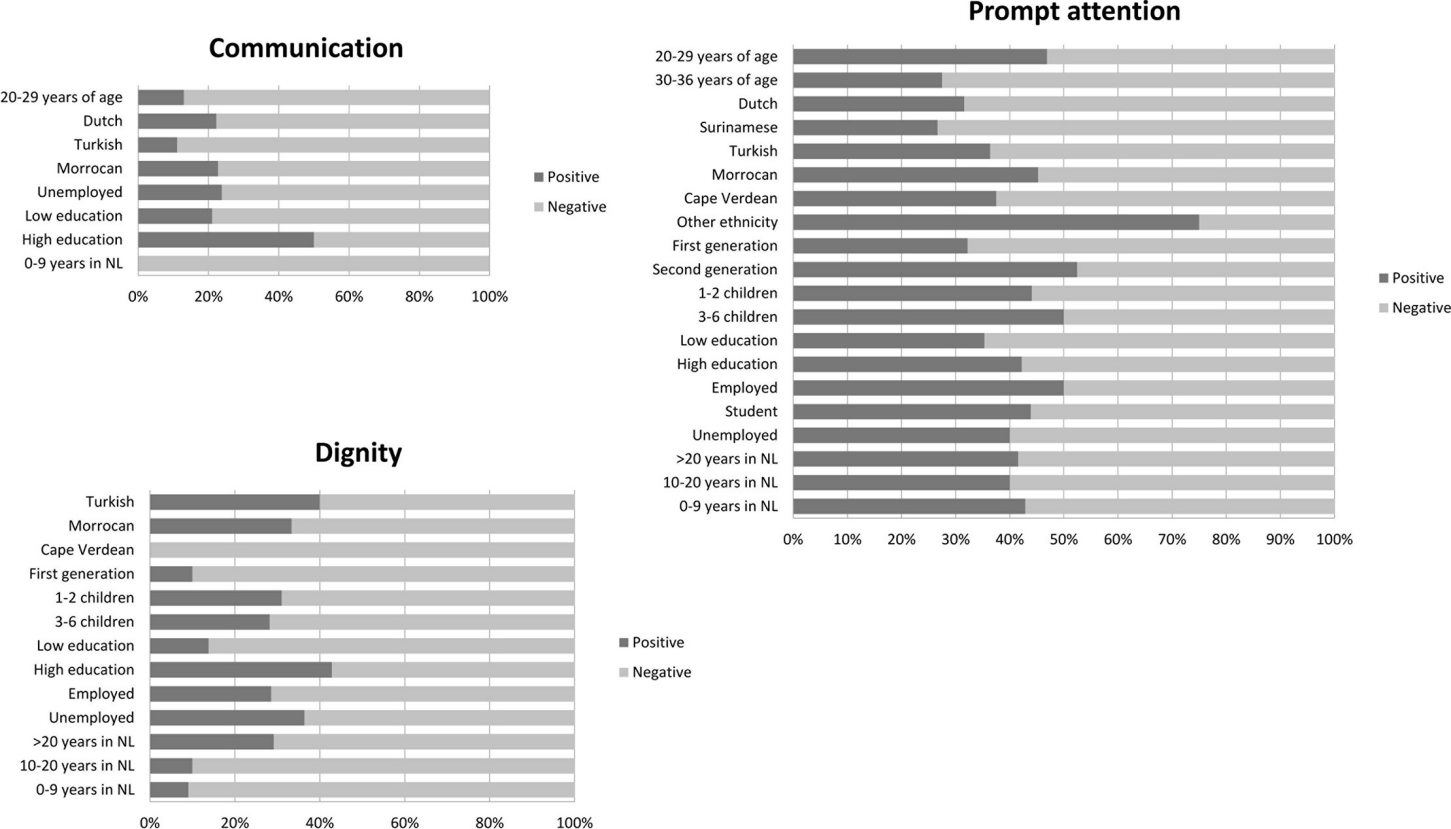
Revelant responsiveness domains in birthing care. If the majority of the respondents voiced their opinion on a specific domain, the results (either positive or negative) were presented in this figure. Positive: a quote was defined as positive when words such as “good”, “adequate”, “nice” and “sufficient” were used. Negative: a quote was interpreted as negative when words such as “bad”, “inadequate”, “not sufficient” and “not nice” were used.

**Fig 4:**
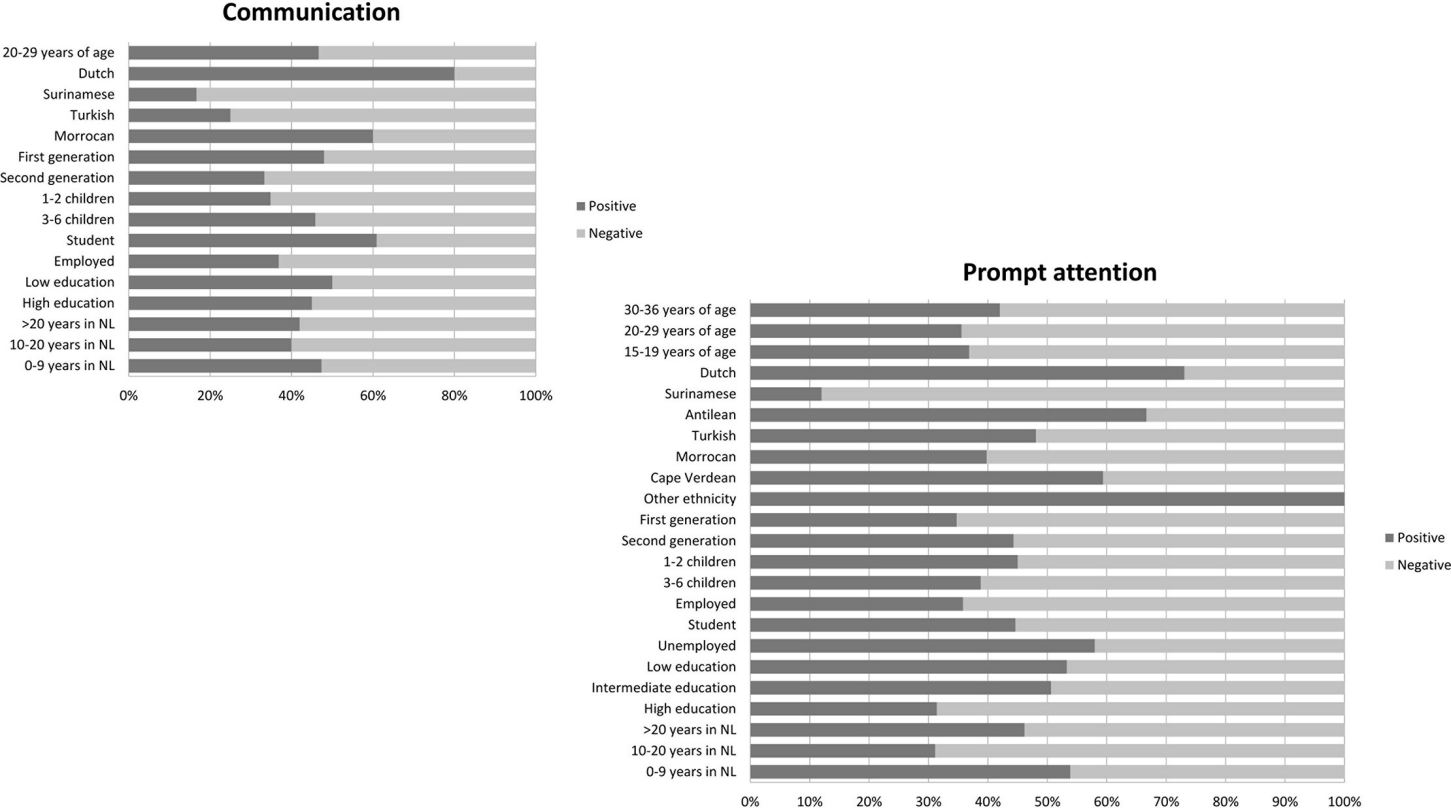
Revelant responsiveness domains in maternity care. If the majority of the respondents voiced their opinion on a specific domain, the results (either positive or negative) were presented in this figure. Positive: a quote was defined as positive when words such as “good”, “adequate”, “nice” and “sufficient” were used. Negative: a quote was interpreted as negative when words such as “bad”, “inadequate”, “not sufficient” and “not nice” were used.

We succeeded in recruiting 106 women with a low level of education or a non-western ethnic minority background (Table 1). If the majority of the respondents voiced their opinion on a specific WHO responsiveness domain the results (either positive or negative) were presented in Figs 2, 3 and 4 and a contextual description of these results was given in the results section.

### Respect for persons responsiveness domains

The respondents expressed opinions about the freedom of decision making and the communication with the caregiver during antenatal care. These opinions were related to the level of satisfaction with antenatal care concerning the domains ‘*autonomy’* and *‘communication’* (see Fig 2). Regarding birthing care the respondents had the most dominant views about the domains ‘*communication’* and ‘*dignity’* (see Fig 3). Regarding the experienced maternity care, the respondents felt most strongly about the *‘communication’* domain (see Fig 4).

#### Autonomy

***In antenatal care*:** Women who had lived in the Netherlands for a longer period of time (more than 20 years), women with a second-generation ethnic minority background and women who were employed had a more positive experience of *‘autonomy’*. They underlined the possibility to freely make their informed decision about participation in physical consultation and antenatal screening. These respondents referred to autonomy in healthcare as *the rights of a pregnant woman*.

Negative experiences in ‘*autonomy’* were mainly related to the lack of informed decision making about antenatal screening and were mostly reported by respondents between 20–29 years of age and respondents living 0–9 years in the Netherlands. A part of these respondents had not received information about first trimester antenatal screening and were not asked if they wanted to participate in this screening. For several respondents this felt like a limitation in their care and treatment opportunities and they did not understand why it happened to them. Some of the respondents suspected there was some relation with their religion or the fact that they were veiled. In other cases, the lack of autonomy seemed more related to suboptimal information provision by the caregiver. The following quote illustrates this as well as the consequence of the need to pay for a test on informed decision making:

*“Well*, *I didn’t get real counselling [about first trimester antenatal screening] because I thought that I knew everything*, *because I underwent the screening in my first pregnancy and then I received the information*. *During this pregnancy I haven’t* ***considered it anymore*, *also because I heard the amount of money that the screening costs*. *You can tell me what y****ou want*, *but I cannot afford it anyway*.*”*

Susan, 33 years of age, Dutch

#### Communication

***In antenatal care*:** specifically amongst Dutch, Cape Verdean and unemployed respondents, positive experiences with the clarity of explanation about prenatal examination, antenatal screening and the encouragements and time to ask questions, led to a more positive opinion about *‘communication’* in antenatal care in general. Mainly native Dutch respondents felt that they could ask questions any time during consultations and if necessary by phone as well. A part of the Cape Verdean and unemployed respondents indicated that they had received extensive information (verbally and written) about antenatal screening and the birthing process. This was experienced as adequate information provision. Negative experiences with the given verbal explanations about the available antenatal care, timing of information transfer, understandability of flyers and the encouragements and available time to ask questions were reasons for Surinamese, Turkish and the first-generation immigrant respondents to have a negative judgment about *‘communication’* in antenatal care. Surinamese respondents were particularly negative about the inconsistency in the information provided on what is healthy and harmful during pregnancy, the listening skills of the caregiver, frequent use of medical terms and the directive communication about, for example, the risks of the cultural use of a specific type of wine. The Turkish and first-generation immigrant respondents experienced communication difficulties and a lack of information also due to the frequent use of medical terms, complex leaflets and websites, absence of information provision in higher parity women and barriers in having a relative present at consultation as an interpreter.

Next a quote of a woman who was pregnant of her fourth child and who received less information:

*“Yes*, *I say honestly during this pregnancy they took a quick look at the baby*, *measured it*, *checked if everything was ok and in the meantime*, *they did not say much*. *While during the first pregnancy that I experienced*, *I found it was perfect*. *They asked if it was my first pregnancy and they explained everything very well with every action like ultrasounds*. *But now they knew it was my fourth pregnancy and automatically they do not tell anything*.*”*

Fatma, 28 years of age, second generation Moroccan immigrant background

***In birthing care*:** concerning birthing care, all subgroups had a negative opinion about ‘*communication’* responsiveness. Women in all subgroups were most negative about the understandability of provided information by the obstetric caregiver about the phases in the birth process and medical interventions during labour, the use of medical jargon and the absence of continuity in the given information *(subdomain ‘understandability of verbal information’)*. Additionally, the majority of the respondents were of the opinion that only when you raised your voice you were heard. Especially the respondents from the Turkish and ‘20–29 years of age’ subgroup judged the communication during labour as inadequate. These groups gave examples of insufficient listening to the childbearing woman, lack of communication about the progress of labour and additionally the ‘20–29 years of age’ group experienced the information about the executed instrumental delivery as inadequate. The following quote illustrates the absence of adjusted communication during labour:

*“I am experienced at giving birth*, *because I already have two children and I felt that the baby was coming*. *There was an intern next to me and she said over and over again*: *“It is not even ten o’clock*, *the baby is not coming”*. *Then I said to her*: *“I have not made a specific appointment with the baby; the baby is coming now*!*” The baby wants to come out*, *I cannot wait*. *I have to push*.*”*

Meryem, 37 years of age, first generation Turkish immigrant background

***In maternity care*:** the native Dutch group was most positive about communication during the provision of maternity care. This was mostly due to adequate explanations about the tasks of the maternity nurse and advice on lactation, bathing of the new born and bedtime rituals. All subgroups pointed out issues in the understandability of verbal information. This included discontinuity and a lack of information, absence of transparent interaction and incomprehensibility of verbal explanations and written materials *(subdomains*: *Understandability of verbal information’ and ‘Understandability of written information’)*. The Surinamese and Turkish groups felt most negative about *‘communication’* in maternity care. Some Surinamese respondents noted that maternity nurses communicated too loudly. Moreover, they considered the given verbal information concerning care for mother and child as inadequate. They had this opinion because the given information did not meet the Surinamese cultural beliefs and rituals (binding and massaging of the new mother’s belly and use of herbs) during the postpartum period. Turkish respondents were mainly negative as a result of experienced lack of written information about the maternity care and the absence, confusing or limited verbal information about child care and breastfeeding. The following quote exemplifies lack of adjusted communication and a lack of transparent communication between the new mother and the maternity nurse.

*“You do not want to be rude*, *because you just gave birth and she comes to help you out*. *You cannot ask to be more quiet because I want to sleep*. *Finally*, *I asked her to go away*. *I had to do so because she was too enthusiastic […] but look at the situation from my point of view*, *please*. *Try to feel how it is to have stitches*.*”*

Emine, 27 years of age, second generation Turkish immigrant background

#### Dignity

***In birthing care*:** in general, the focus group respondents were negative about the dignity domain in birthing care. Disrespect in terms of verbal and non-verbal interaction towards the woman giving birth and her social network was the reason mentioned most often *(subdomain ‘Respect from the healthcare provider’)*. Within all subgroups there was also a negative opinion about the frequent examinations during labour by too many different caregivers. The respondents experienced this as disrespectful. These experiences occurred in the secondary and tertiary care settings. The Cape Verdean group, the first-generation immigrant group and the low-educated respondents were most negative. The Cape Verdean respondents were particularly negative about the lack of privacy in the delivery room and the disrespectful reactions of caregivers on expressions of pain by the childbearing woman. A part of the first-generation respondents experienced loneliness during labour. They had the feeling that their needs were not being taken seriously. These respondents were also confronted more often with agitated caregivers because of undesirable behaviour by the childbearing woman or her relatives (e.g. too many family members at the bedside). Low-educated respondents experienced partly the same aforementioned negative experiences, but also reported the absence of a specific caregiver whose presence would have been preferred by the childbearing woman.

The next quote presents an example.

*“When I was in labour*, *my water broke and then an intern or doctor came in*. *She said it was disgusting*. *That was bad of her*, *it was painful*, *I was really grieved because I could not help it*. *I was afraid to say anything*, *it was too painful and too shameful and then you do not say something like that*.*”*

Jamilah, 31 years of age, first generation Moroccan immigrant background

Concerning antenatal care, respondents had no clear opinion about the ‘respect for persons’ responsiveness categories *‘Confidentiality’* and *‘Dignity’*. In birthing care the respondents had no dominant view about the *‘Autonomy’* and *‘Confidentiality’* domains. Concerning the satisfaction with maternity care, the domains *‘Autonomy’*, *‘Confidentiality’* and *‘Dignity’* did not play an important role in the experience of the respondents

### Client orientation responsiveness domains

The way in which care was provided and the timing of care during all phases of pregnancy, labour and childbed, was experienced as most important by all women. This opinion was related to the level of responsiveness concerning the WHO domain ‘*prompt attention’* (see Figs 2, 3 and 4). This domain falls under the responsiveness category *‘client orientation’*.

#### Prompt attention

***In antenatal care*:** most of the quotes of the Antillean and the low-educated respondents showed satisfaction with the adequateness and quickness of the provided antenatal care. Thus, these groups were mostly positive about the prompt attention domain in antenatal care. Antillean respondents mainly based their positive opinion on the adequate recognition of medical risks during pregnancy and swift referral by a midwife (primary care) to the obstetrician in the hospital (secondary or tertiary care). The low-educated respondents were mainly positive about the prompt attention of the provided antenatal care by midwives in terms of the ease in making appointments, frequent consultations and the cosy atmosphere in their practices. Women of more than 36 years of age and those who lived more than 20 years in the Netherlands were the most negative about *‘prompt attention’* in antenatal care. More specifically, a part of the women aged over 36 found antenatal care provision of low quality, particularly in primary care. The also mentioned discontinuity in the caregivers they met during their pregnancy. Respondents who had lived for more than 20 years in the Netherlands were especially negative about the late timing of the first antenatal visit by antenatal healthcare organisations, poor accessibility of healthcare locations and the lack of knowledge and professionality of antenatal caregivers. This lack of knowledge pertained to risks of specific drugs in pregnancy and symptoms related to pregnancy.

The next quote is an example of the discussion about the adequateness of prompt attention during antenatal care by the pregnant women:

*“They heard what I said, but they do not take me seriously. I discussed my annoyances about it and they changed. The next appointment was not after 4 weeks, but after 2 weeks and they asked me how I felt*. *It was nice, but I thought: why didn’t you ask that earlier in my pregnancy?”*

Choukria, 30 years of age, first generation Moroccan immigrant background

The following quote is an example of lack of professionality and attention:

*“As a healthcare professional you must know what to do with special patients [above 40 years of age and cardiovascular disease in the family]*. *They acted insecure*. *I have met two midwives*, *but with one of them I sensed a lack of professionalism*, *no passion for her profession*. *She was not involved and acted pragmatic*. *She only handed over stencils and I saw that she was in a hurry*. *I missed her attention; it is all about an unborn child*, *a new life*. *It is not about buying new furniture*! *You must be a professional and give care with your heart*.*”*

Josipa, 40 years of age, first generation Bosnian-Croatian immigrant background

***In birthing care*:** women in the ‘other ethnicity’ and second-generation groups were the only ones who were predominantly positive about the ‘prompt attention’ in terms of quickness, sufficiency and professionality of the birthing care, especially when provided in a hospital setting. Within all other subgroups the timing and the appropriateness of the provided birthing care were judged negatively *(subdomains*: *‘Prompt attention of healthcare service’ and ‘Waiting time after asked for help’)*. Examples include long waiting times to receive care during an emergency situation, inconsistency in the provided care, discontinuity in healthcare professionals and not being able to find birthing care professionals as a result of changing shifts. The respondents’ quotes indicated that social support during labour contributed to the childbearing woman’s experienced prompt attention in birthing care. The following quotes show the perceptions of two respondents about ‘prompt attention’ in birthing care.

*“Experiences with the provided care are*, *to my opinion*, *not influenced by the hospital system*, *but the individual healthcare professionals you meet during labour*.*“*

Virginia, 46 years of age, second generation Cape Verdean immigrant background

*“It did not go well…*. *I had a little blood loss at home and I went to the hospital and in the hospital*, *I waited and screamed*. *A nurse heard me and comforted me a little bit and said that the doctor would take a look at me*. *[…] The doctor came in and acted immediately*. *In the operating room the nurse told me that my child had passed away*. *That was terrible for me*. *I didn’t know what happened*.*”*

Zahrah, 35 years of age, first generation, Moroccan immigrant background

***In maternity care*:** the Dutch, Antillean and ‘the other ethnicity’ groups were most positive about this domain in maternity care. Examples of these ethnic groups’ positive experiences with prompt attention were stories about maternity nurses who provided exactly the care and psychosocial support needed. As examples of negative experiences in terms of prompt attention, women often mentioned a strict focus on the new born and a lack of attention and involvement of maternity caregivers with the new mother. This included care being provided too late or not at all, and unprofessional behaviour *(subdomain*: *‘prompt attention of healthcare service’ and ‘waiting time after asked for help’)*. Furthermore, women gave examples about the discontinuity of provided maternity care (‘choice of care provider’ domain). Notably, maternity care provided in hospital was judged more negatively than was maternity care at home.

The Surinamese and highly educated women were negative about the prompt attention domain. The Surinamese respondents experienced that maternity nurses were not knowledgeable enough to provide adequate care and to be of added value during the postpartum period. Furthermore, a number of these respondents felt that they were more knowledgeable and capable in caring for themselves and their new born than the maternity nurse. Highly educated respondents had the same opinion about the low level of knowledge and lack of added value of maternity nurses. They found maternity care especially in the hospital setting inadequate. Specific issues mentioned included insufficient time to rest for the new mother and her new-born, and insufficient recognition of postpartum risks. The following quote is from a respondent who experienced that maternity care underserves the new mother:

*“They (professionals at the maternity department) see new mothers not as new mothers*. *It is only the new-born that matters; that is their patient*. *The new mother should be grateful that she can stay in the hospital*.*”*

Aisha, 33 years of age, first generation Moroccan immigrant background, intermediate educational attainment

The respondents in the focus group interviews did not give a clear opinion about the ‘client orientation’ responsiveness category domains *‘Quality of basic amenities’*, ‘*Choice of care provider’* and *‘Access of social support’*.

## Discussion

### Key findings

Overall, when comparing the different phases of care, women were most positive about the care they received during the antenatal phase. However, this study showed that generally, respondents with a non-Western ethnic background and low education had a negative opinion about Dutch health care responsiveness during all phases of antenatal, birthing and maternity care.

The findings concerning the ‘*communication’* domain in all three phases of care are in line with research by Degrie et al. These researchers conducted a systematic review of qualitative research with a focus on healthcare provision to immigrants and the user’s perspective on the received care.[45] In the present study the negative judgment on ‘*communication’* especially by first generation non-western ethnic minority women seems to spring from their low-educational attainment and limited Dutch language proficiency level. Previous studies have demonstrated that these maternal factors play an important role in this negative sentiment. These maternal factors are related to communication problems such as (1) cultural differences in explanatory models of health and illness, (2) differences in cultural values, (3) problematic or conflicting relations between acculturation orientations of caregivers and patients, and (4) linguistic barriers.[46, 47]

The negative judgment of ‘*prompt attention’* by non-western and low-educated respondents was consistent with other research in the field.[16, 48] These studies show that reasons for care utilization problems and the negative view on obstetric care include inappropriate timing and incompatible opening hours, transport and mobility problems, indirect (unintentional) discrimination and caregivers’ lack of knowledge about cultural practice.

### Discussion of ‘respect for persons’ responsiveness domains

For the respondents in our study, the ‘respect for persons’ domain of *‘confidentiality’* had no importance in either a positive or negative sense. In previous quantitative studies, this domain performed better than other domains and was judged to be more important than the other domains within the category ‘client orientation’ (also see Box 1). [27, 49] Possible explanations for these differences in study outcomes are the differences in used study designs. The aforementioned studies used questionnaires and structured interviews, which required participants to rate the ‘confidentiality’ domain. Also, our study population differed from that in other studies, in terms of a higher number of women with a non-western ethnic background and low educational attainment.

Our study shows that the performance of *‘autonomy’* in antenatal care was experienced more positively by respondents that have lived in the Netherlands for a longer period of time, had a second immigrant generation background and were employed. The respondents in our study who had migrated to the Netherlands only recently, experienced decreased *‘autonomy’* in antenatal care. This is in line with prior studies. [15, 16, 50, 51] A longer duration of residence in the Netherlands may have led to better acculturation and greater chances of employment. This impression is consistent with previous studies demonstrating that a higher socioeconomic status of non-western immigrants resulted in more *‘autonomy’* in the utilization of antenatal care. [15, 16, 50, 51] It seems that a higher level of acculturation, which positively correlates with socioeconomic status, contributes to the *‘autonomy’* aspect in the use and provision of obstetric healthcare.

The results of the present study indicate that respondents with Dutch or Cape Verdean ethnicity and those who were unemployed experienced *‘communication’* as more positive than did other respondents. For the Dutch respondents this can be explained by their language proficiency and their knowledge about the Dutch healthcare system. Nevertheless, Cape Verdean women have lower levels of self-reported Dutch language proficiency.[52] Being unemployed and having low educational attainment are conditions associated with health literacy issues.[15] However, unemployment as an individual risk factor does not have to lead to interaction difficulties with healthcare providers. Prior studies demonstrated that having more time for health care appointments due to unemployment can have a positive effect on interaction with healthcare *providers*.[53, 54]

Based on our study findings, the communication aspect in the provision of Dutch obstetric care is not suitable for women with a first-generation non-western immigrant background (especially Surinamese and Turkish). Several previous studies have also underlined the strong association between a first generation background and communication problems in obstetric care.[15, 16] Our findings for the Turkish women were consistent with previous research that demonstrated a negative association with having a first generation Turkish immigrant background and having to communicate about medical topics with the caregiver.[55] Literature provided no confirmation for the findings of the Surinamese group concerning the inadequate communication in obstetric care. Possibly their negative opinions were influenced by the caregivers’ directive and culturally unadjusted communication skills and the fact that the content of given information did not meet their cultural and religious views on health and healthcare. [56]

Our study results indicate that the non-western (especially Cape Verdean) respondents and low-educated respondents perceived a lack of respect in obstetric care, particularly during labour. Previous studies confirm that women with a non-western immigrant background perceived a lack of respect from the healthcare system whilst they were giving birth. [57, 58] This inadequate fulfilment of *‘dignity’* is in line with prior studies that show that especially women with a low socioeconomic status background had to deal with a lack of respect from obstetric caregivers during labour. [58]

It seems that the current obstetric healthcare system in the Netherlands is insufficiently capable to act in a culturally competent manner, resulting in inadequate cross-cultural communication. The negative view of the second-generation immigrant respondents concerning the communication component in particularly maternity care, is in concordance with previous studies conducted in the Netherlands and United Kingdom.[8, 59]These studies described a lack of knowledge about the concept of maternity care and postpartum health by immigrant women (first and second generation) as a reason for interaction difficulties in the provision of maternity care.

### Discussion of ‘client orientation’ responsiveness domains

The focus group respondents judged the ‘client oriented’ responsiveness domains *‘quality of basic amenities’*, *‘choice of care provider’* and *‘access of social support’* to be unimportant. The lack of importance of three of the four domains in this category is in line with the findings of another study about the quality of the obstetric care system. In that study the ‘client orientated’ category was found less important than the ‘respect for persons’ domains.[27]

In the present study, prompt attention (which falls in the ‘client oriented‘ category) did heavily influence the experience of responsiveness of the Dutch obstetric care. The more acculturated respondents and the low-educated respondents experienced *‘prompt attention*’ in obstetric care most positively. This is due to better health literacy and financial resources within the more acculturated groups and lower expectations about the provided obstetric care within low-educated groups. These aspects makes it easier for the healthcare system and its caregivers to provide adequate care to these groups. [15, 60–64] This association was also found in another study concerning factors affecting patient satisfaction.[65] People with higher levels of education are more health conscious, more aware of their consumer rights, ask more questions and more often experience prompt attention in care. [53, 66]

Our negative finding concerning *‘prompt attention’* in obstetric care for respondents who had lived for more than 20 years in the Netherlands is in contrast with other research.[66] These women have higher levels of health literacy and therefore may have higher expectations about obstetric care. [15, 16] Specifically the Surinamese negative views on ‘prompt attention’ in obstetric care may be linked with the Surinamese perception of pregnancy, the birth process and care for new borns, which is influenced by traditional medicine, culture and religion. [56] Prior studies showed a decreased use of antenatal care by multiparous Surinamese-Creole women in comparison with other ethnicity groups. In our study population the Surinamese respondents had a Creole background.[17, 67] The aforementioned study describes a higher cumulative risk for preterm birth within this ethnic group and therefore more provision of urgent and high risk care, which influenced the expectations about the prompt attention in obstetric care.

### Strength and limitations

The representation of a wide range of age groups (from adolescents to (grand)mothers) is an important strength of our study. Consequently, it was possible to gain insight in the different views on the satisfaction with Dutch antenatal, birthing and maternity care from the perspective of various generations and life phases. Other benefits of the study were the large study population and the fact that for most of the background characteristics data saturation (the point when incoming data produces little or no new information) was reached.[28, 29] Recruitment of focus group members by using active recruitment methods was appropriate for our study, because non-western immigrants and low-educated are frequently underrepresented in research. The focus group method was useful for these target groups, because they more frequently experience barriers to filling in questionnaires due to inadequate language proficiency and illiteracy. [28, 68, 69] Furthermore, the use of this method made it possible to execute several coding steps and a more in-depth analysis based on background characteristic.[27, 28]

Still, several limitations need to be addressed. First, despite interpreting by one of the moderators, field experts and other respondents in the focus groups for respondents with a lack or limited language proficiency level, some respondents may not have well understood all the questions. Second, it was not possible to check to what extent the respondents’ quotes about experienced antenatal, birthing and maternity care were influenced by the group dynamic (peer pressure) during the session. Because the focus groups consisted of a number of participants by design, the influence of peers on the views expressed by women cannot be ruled out. This is a difficult trade-off between the benefits and disadvantages of this form of research design. Third, although there was no specific difference in outcomes of the focus group sessions led by the different moderators, a possible influence of these professionals on the focus group results cannot be ruled out. Fourth, during the course of the study we measured the level of data saturation only superficially. During data analysis, however, the level of data saturation was found adequate. [28, 29]

### Policy recommendations

Based on our study results and the known health inequities in between the group of low-educated native Dutch and non-western immigrants and the group of native Dutch and higher educated women, there is a need to provide more extensive care to the former group of, who already are vulnerable. The inequalities in responsiveness of the obstetric healthcare for non-western and low-educated women as observed in this study urges for improvement, in particular as these inequalities are in part responsible for the perinatal outcome gap in the Netherlands.[1,6] Since responsiveness of the healthcare system is getting more important and our study underlines the inadequateness of the responsiveness domains *‘autonomy’*, *‘communication’*, *‘dignity’* and *‘prompt attention’* in obstetric care for vulnerable women, we recommend focussing on cross-cultural communication and improvements in cultural competence in the obstetric healthcare system. Providing care that is adjusted to the pregnant women’s and new mothers’ cultural preferences starts with communicating with the women about their cultural history, their needs for health information on the basis of which they can make informed decisions in healthcare and identify their care goals.[70, 71] The domains of *‘autonomy’*, *‘dignity’*, and *‘communication’* can be hard to change, because these are influenced by professionals’ behaviour and can only be changed by self-reflection, intervision and training, which requires time and support of the obstetric caregivers. Changing the ‘client orientation’ domain *‘prompt attention’* requires organizational changes in terms of management coordination, whose implementation may take less time. Therefore, we advise to focus first on the ‘respect for persons’ domains and recommend the health educational institutes and obstetric healthcare organisations to integrate education about cross-cultural communication and cultural competence into the training of obstetric caregivers. The training should aim at increasing the obstetric caregivers’ insight and knowledge concerning: (1) how patients experience pregnancy, childbirth and the postpartum period and received care based on the influence of culture; (2) the elements of cross-cultural communication (self-awareness and respect for the pregnant women’s and new mothers beliefs and values); and (3) cultural competent healthcare provision (attention for cultural and religious beliefs of patients and cultural sensitive attitude of the caregiver). Furthermore, we recommend the Dutch local governments to facilitate peer health education programs focusing on the Dutch healthcare system and reproductive health. Health-related peer education is an approach whereby community members are supported to promote health-enhancing change among their peers. Conditional for implementation of aforementioned recommendations are incentives of the Dutch government and health insurance parties to motivate health educational institutes, obstetric healthcare organizations and caregivers to take up these recommendations.

### Future research

In view of the study outcomes it would be relevant to repeat the focus group study exclusively with multi-ethnic, low-educated pregnant women and women who recently gave birth, so as to explore their recent experiences with the responsiveness of the antenatal healthcare system. Such a prospective study design would reduce the risk of recall bias. [72] In this future research it would also be interesting to compare characteristics of health care services in the countries of origin with the Dutch health care services. It would then be possible to elucidate whether this explains part of the negative perceptions of women. Moreover, in a future study it would be helpful to ask women to elaborate on how they believe health care could be improved to meet their needs.

## Conclusion

The study population was mostly positive about the provided care during the antenatal phase. *However*, this study shows that the healthcare system responsiveness of the Dutch obstetric healthcare system in all phases of care (antenatal, birthing and postpartum) does not meet the needs of the non-western and low-educated women in our study population. These women experience decreased *‘autonomy’*, inadequate *‘communication’* and insufficient *‘prompt attention’* in obstetric healthcare. The study findings give contextual meaning and clues for diminishing responsiveness inequalities in the provision of obstetric care within a multi- ethnic women’s population.

## Supporting information

S1 Table Interview protocol–focus group interviews. ^1^ Subjects of the follow-up questions, formulation of the question and the moment of asking were based on the focus group session dynamics, individual answers and the group consensus.
(DOCX)
