# Supplementary material for: Satisfaction with obstetric care in a population of low-educated native Dutch and non-western minority women. Focus group research
Source: PLoS One. 2019 Jan 31;14(1):e0210506. doi: 10.1371/journal.pone.0210506 (PMC6354976; doi:10.1371/journal.pone.0210506)
Supplement: S1 Table — 1 Subjects of the follow-up questions, formulation of the question and the moment of asking were based on the focus group session dynamics, individual answers and the group consensus. (DOCX) [file pone.0210506.s001.docx]

**S1 Table 1. Interview protocol – focus group interviews**

| **Structure** | **Action** | **Information / questions** |
| --- | --- | --- |
| *INTRODUCTION* | Information | - Study information  - Structure of the session  - Use of collected data  - Identifying information of respondents will not be transcribed  - Aim of the session: obtaining their experiences and satisfaction with antenatal, birthing and maternity care |
|  | Question | *Do you give permission for the recording of this conversation?* |
| *PART I*  Antenatal care | Engagement question | *How do you experience Dutch healthcare during pregnancy?* |
|  | Exploration questions^1^ | Based on the domains of responsiveness of care as shown in box 1.  Chronology to discuss domains: *Autonomy, Choice of care provider, Communication, Prompt attention, Dignity, Access of social support, Quality of basic amenities.* |
|  | Exit questions | *What is adequate care during pregnancy?*  Are there other things that you want to tell about the experienced antenatal care? |
| *PART II*  Birthing care | Engagement question | *How did you experience Dutch healthcare during labour?* |
|  | Exploration questions^1^ | Based on the domains of responsiveness of care as shown in box 1.  Chronology to discuss domains: *Prompt attention, Dignity, Choice of care provider, Communication, Access of social support, Autonomy, Quality of basic amenities.* |
|  | Exit questions | *What is adequate care during labour?*  Are there other things that you want to tell about the experienced birthing care? |
| *PART III*  Maternity care | Engagement question | How did you experience Dutch healthcare during the postpartum period? |
|  | Exploration questions^1^ | Based on the domains of responsiveness of care as shown in box 1.  Chronology to discuss domains: Communication, *Prompt attention, Access of social support, Autonomy, Dignity, Quality of basic amenities, Choice of care provider.* |
|  | Exit questions | *What is adequate care during the postpartum period?*  Are there other things that you want to tell about the experienced maternity care? |

^1^ Subjects of the follow-up questions, formulation of the question and the moment of asking were based on the focus group session dynamics, individual answers and the group consensus.
